# Can housing improvements cure or prevent the onset of health conditions over time in deprived areas?

**DOI:** 10.1186/s12889-015-2524-5

**Published:** 2015-11-28

**Authors:** Angela Curl, Ade Kearns

**Affiliations:** Urban Studies, University of Glasgow, 25 Bute Gardens, Glasgow, G12 8RS UK

**Keywords:** Housing improvements, Health conditions, Deprived areas, Glasgow UK

## Abstract

**Background:**

There is a need for more evidence linking particular housing improvements to changes in specific health conditions. Research often looks at generic works over short periods.

**Methods:**

We use a longitudinal sample (*n* = 1933) with a survey interval of 2–5 years. Multivariate logistic regression is used to calculate the odds ratios of developing or recovering from six health conditions according to receipt of four types of housing improvements.

**Results:**

Receipt of fabric works was associated with higher likelihood of recovery from mental health problems and circulatory conditions. Receipt of central heating was also associated with higher likelihood of recovery form circulatory conditions. No evidence was found for the preventative effects of housing improvements.

**Conclusions:**

Health gain from housing improvements appears most likely when targeted at those in greatest health need. The health impacts of area-wide, non-targeted housing improvements are less clear in our study.

## Background

This paper examines the direct pathway between housing improvements and health conditions in Glasgow, UK, a city with a history of poor public health [1]. While there is a long history of association of housing conditions and health [2, 3] the evidence linking housing improvements with health outcomes is more sparse. Furthermore, while some studies have evaluated housing or area renewal programmes as a whole, there is less evidence for understanding the effects of specific housing improvements on particular health conditions. Some evidence suggests that in order to achieve positive health outcomes housing improvements are best directed towards those with poor health [4, 5], but it is also worthwhile exploring whether housing improvements can prevent the onset of certain health conditions amongst a relatively deprived population at greater risk of poor health.

The GoWell study, which we report on here, explores the impacts of investment in housing, regeneration and neighbourhood renewal on the health and wellbeing of individuals, families and communities in deprived areas alongside a ten year programme of investment in its housing stock by the largest social landlord in the city, Glasgow Housing Association (GHA): around 45,000 properties were to be improved from 2003–2015. Against a backdrop of continued high relative mortality rates in the city [1, 6–8] and rising morbidity in our study population [9], we investigate whether improvements in housing conditions can prevent or cure a number of health conditions commonly associated with poor housing quality.

We have previously examined changes in general physical and mental health outcomes related to housing improvements using the SF-12 component scores and found positive associations between fabric works and physical and mental health, and between new kitchens and bathrooms and new front doors with mental health, but negative associations between central heating and mental health [10]. Here, the purpose is to explore the effects of housing improvements on specific self-reported health conditions which we expect to be affected by relevant aspects of housing quality. In the recent Cochrane review [4] studies were categorised into warmth and energy efficiency improvements or retrofitting and neighbourhood renewal [4]. Here we study specific housing interventions as part of a wider retrofitting and neighbourhood renewal programme. We investigate four particular housing improvements based on availability of data from GHA: fabric works; new kitchens, bathrooms & rewiring; new front doors; and provision of central heating. The content of these works is described in Table 2.

The majority of available evidence on the effects of housing improvements relates to respiratory health conditions and is sparse in relation to other health conditions. Previous studies have found significant effects when housing interventions are targeted at those with pre-existing health conditions and this is the recommendation of the Cochrane review [4]. For this reason we took a sub-sample of those with pre-existing health conditions to assess whether those who receive housing improvements show any signs of health improvement over the period.

It is also feasible that rather than leading to recovery from long-term health conditions over a relatively short period, housing improvements serve to mitigate against the onset of poor health in a relatively deprived population. Therefore, taking as the sample those not reporting health conditions at first interview, we predict the odds ratios for reporting that condition at second interview according to receipt or not of housing improvements. In studies reviewed by Thomson et al. [4] the follow-up interval was often one year or less, with the longest time post intervention being 3.5 years in a study by Shortt and Rugkåsa [11], however we interview householders up to 5 years after the intervention.

The aim of this paper therefore, is to establish whether specific housing improvements are related to the amelioration or onset of certain self reported health conditions, based on the conceptual framework of causal pathways outlined below.

### Health risk factors and housing conditions: conceptual framework

In this section we identify which housing improvements are likely to affect particular causes of ill-health so as to conceptualise which housing improvements might impact each health condition and present evidence from past studies, where available. Table 1 summaries the theorised pathways between housing improvements and health conditions (arrow denotes direction of relationships) based on housing-related risk factors for each health condition, which are discussed in turn below.

**Table 1:** Pathways from housing improvements to health conditions

			
Health condition	Housing risk factors	Associated housing conditions	Relevant housing improvements
Respiratory health	Damp and mould.	Thermal efficiency	Fabric works
	Cold.	Weatherproofing	Central heating
	Overcrowding	Ventilation	Kitchens & bathrooms
Circulatory conditions	Cold.	Thermal efficiency	Fabric works.
		Heating affordability and functioning.	Central heating.
Digestive health	High-blood pressure.	Standard of food storage, preparation and cooking facilities.	Kitchens and bathrooms.
	Unhealthy diet.		
Allergies & skin conditions	Dust mites.	Thermal efficiency	Fabric works
	Damp and mould.	Weatherproofing	Central heating
	Infestations.	Heating affordability and functioning.	Kitchens & bathrooms
	Soft furnishings.		
	Cleaning.	Quality of work and floor surfaces.	
	Temperature and humidity.		
Headaches and migraines	Stress.	External quality and appearance.	Fabric works
	Anxiety.		Central heating
	Depression.	Damp and mould	Kitchens and bathrooms
		Fuel poverty	
		Overcrowding	New front door
		Concerns about crime and antisocial behaviour	
Mental health	Stress.	External quality and appearance.	Fabric works
	Anxiety.		Central heating
	Depression.	Damp and mould	Kitchens and bathrooms
		Fuel poverty	
		Overcrowding	New front door
		Concerns about crime and antisocial behaviour	

#### Respiratory conditions

Our measure of respiratory conditions includes breathing problems, asthma or bronchitis. The main housing-related risk factors for these conditions are damp and mould [12–15], cold homes, pollutants, infestations and overcrowding. Damp and mould are associated with respiratory problems and asthma, whilst indoor pollutants and infestations can trigger asthma [3]. Living in cold homes can diminish resistance to respiratory infections and overcrowding increases the risk of respiratory disease through contagion [3]. We hypothesise that central heating and fabric works, as warmth interventions, will be related to respiratory conditions by improving warmth and reducing damp and mould. Overcrowding may be reduced through central heating improvements by expanding the usable space in the house given improved warmth. Furthermore we assume that new kitchens and bathrooms may eliminate problems of mould and infestations so we also test this relationship.

Previous studies have generally found improvements against a wide range of respiratory outcomes [5, 11, 16–20] related to warmth and energy efficiency works, particularly when targeted at households with inadequate warmth and where at least one member had a pre-existing respiratory condition. Platt et al. [19] and Shortt and Rugkåsa [11] report both positive and negative outcomes. Woodfine et al. [20] found non-significant, improvements in asthma following central heating. Braubach et al. [21] found a small reduction in the proportion reporting common cold and bronchitis, but no effect on asthma. Hopton [22] found fewer reports of persistent cough or runny nose, but higher reports of wheezing. Somerville [23] reports improvements for cough, wheeze, and blocked nose and Iverson et al. [24] found reduced reports of dry throats. Conversely, in studies of retrofitting or neighbourhood renewal, Kearns et al. [25] found a non-significant negative impact on wheezing, Ambrose [26] found negative impacts on coughs, colds, bronchial conditions and Blackman et al. [27] reported a negative effect on acute and chronic respiratory conditions.

#### Circulatory conditions

We define circulatory conditions as relating to the heart, high blood pressure or blood circulation problems. The main housing related risk factor for these conditions is a cold home which is associated with heart disease, heart attacks and strokes [3]. We therefore hypothesise central heating and fabric works as warmth interventions might affect circulatory health conditions.

Two studies have found positive effects on circulatory health, both from Scotland. Lloyd et al. [28] found a statistically significant reduction in blood pressure among a sample of 26 residents, related to a comprehensive warmth intervention consisting of “double skinning walls…insulation, draught proofing, double glazing, gas central heating, solar panels, dual-purpose heat recovery system and inclusion of front and back verandas within the internal living area of the flat”. Walker et al. [29] found a lower probability of reporting a new diagnosis of heart condition among those who had heating compared with those who did not, however they also reported a non-significant negative effect on circulation problems.

#### Digestive conditions

Digestive conditions refer to stomach, liver, kidney and digestive problems. The main risk factors for such conditions relate to diet, including diet-related high blood pressure and unhealthy diets. The relationship between housing conditions here are less clear, but we hypothesise that new kitchens, also free of mould, may encourage more home cooking and healthier diets so therefore test the effect of new kitchens on digestive health. The existing evidence in terms of digestive health is sparse: Howden-Chapman et al. [17] report reduced likelihood of diarrhoea and vomiting in children following warmth interventions. Kearns et al. [25] report no difference post intervention for indigestion whereas Molnar et al. [30] found reductions in reported dietary and digestive problems.

#### Skin conditions and allergies

The main housing risk factors for skin conditions and allergies are dust mites [31], damp and mould [12, 13], infestations, soft furnishings, cleaning, and temperature and humidity. We hypothesise that central heating, fabric works and kitchens and bathrooms affect damp and mould as well as temperature and humidity. Furthermore, new kitchens and bathrooms, which in the Glasgow case usually include new linoleum flooring, may help any problems related to infestations, dust-mites and cleaning. Few studies report effects on skin or allergy conditions, but those that do have found negative results. Kearns et al. [25] found higher reports of eczema in children following improved housing circumstances and Walker et al. [29] found a negative impact of central heating on nasal allergies.

#### Migraines and headaches

The main risk factors for migraines or frequent headaches are stress, anxiety, depression, missed meals and certain foods and drinks. Stress has been related to overcrowding and depression to damp housing conditions. Given the potential for central heating and fabric works to reduce damp and increase the usable space in the house through improved warmth we test the relationships between these improvements and headache conditions. Improvement in energy efficiency through warmth interventions should also reduce fuel poverty, which may also have a positive impact on stress, anxiety and depression. New kitchens and bathrooms can help to reduce the incidence of mould, which is a cause of depression. New front doors are seen as a security improvement which may reduce anxiety about crime and antisocial behaviour, particularly in deprived areas. There is limited evidence relating to migraines or headaches, although Kearns et al. [25] found no differences post intervention.

#### Mental health conditions

We use mental health conditions to refer to long term stress, anxiety or depression.

As described above relating to headaches, we hypothesise that central heating, fabric works, kitchens and bathrooms and new front doors will be related to mental health conditions. Central heating and fabric works may impact upon mental health through their contribution to reducing the energy inefficiency of housing, which affects mental health via the impacts of thermal discomfort [32] and condensation, damp and mould [33]. Energy efficiency improvements through warmth interventions may also have a positive impact on mental health by reducing stress anxiety and depression as a result of fuel poverty; reduction in financial stress has been identified as the most common cause of mental health improvements following energy efficiency works [34]. New kitchens and bathrooms may reduce depression if they eradicate mould, and may also enhance self-esteem where the occupants are given a choice of finishings in order to make the improvements their own.

The majority of studies examining mental health outcomes related to housing improvements have used scale measures of mental health [25, 35, 36]. As outlined above, we have examined this previously [10] and found positive associations with fabric works, but in this paper focus on self reported mental health. Howden-Chapman et al. [5] find better mental health on four measures (SF-12 domains). Woodfine et al. [20] (PedsQL in children) and Barton et al. [16] (GHQ) find a mix of non-statistically significant results. Wells [37] reports a statistically significant improvement in mental health. Allen [38] found improved mental health based on the SF-36 and measures of anxiety. Conversely Ambrose [26] and Blackman et al. [27] report increased stress or depression or mental health issues after housing improvements. Thomas et al. [39] found higher levels of mental distress among the intervention group both before and after the receipt of housing improvements, positing that the former may be due to the effects of living in a house needing improvement, and the latter to disruption caused by the improvement works.

## Methods

### Survey data

**Fig. 1:**
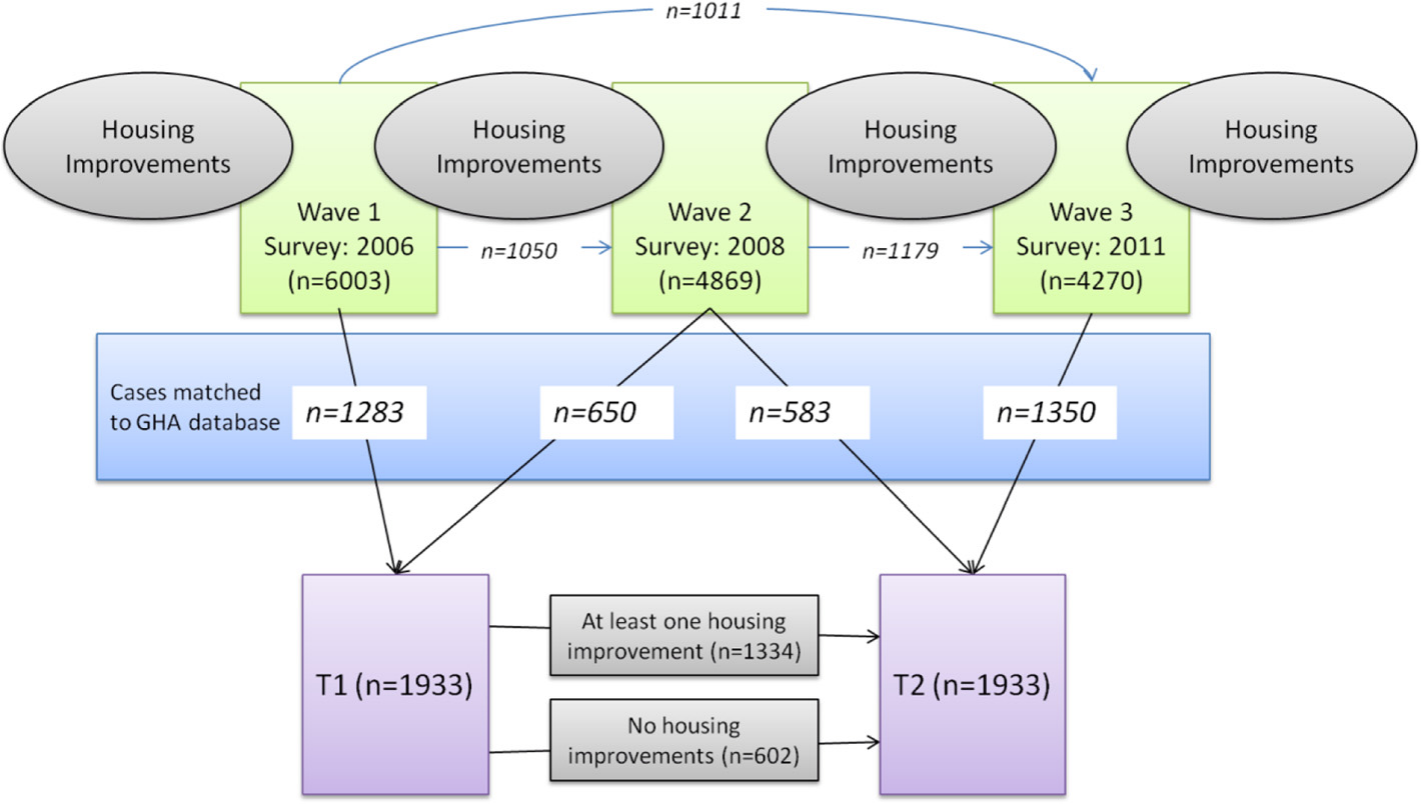
Sample construction. Note: Italics refer to longitudinal cases. Bold and italic are those matched to housing improvement database. Thin (blue) lines indicate the longitudinal subset from which we derive the T1-T2 sample. For example, T2 cases consist of 583 out of 1050 W1-W2 cases and 1350 cases derived either from W1-W3 (1,011) or W2-W3 (1,179)

Our analysis draws upon three waves of a household survey undertaken as part of the GoWell project in Glasgow. We combined the waves into two time periods, T1 and T2 to conduct a before and after intervention study of the effects of specific housing interventions on self-reported health conditions as shown in Fig. 1.

Household surveys were conducted in 2006 (Wave 1), 2008 (Wave 2) and 2011 (Wave 3) using a repeat cross-sectional design with a nested longitudinal cohort. Random samples of addresses across all housing tenures were selected for interview across fifteen study areas in Waves 1 and 2. At Wave 3 all previous addresses where an interview had been conducted were selected for the sample. In six areas where extensive demolition was taking place, all occupied dwellings were selected for interview at each wave, in other areas the overall sample frame was 30 % of all existing addresses at Wave 3, although this varies by study area. The surveys achieved response rates of 50.3 %, 47.5 % and 45.4 %, respectively. Retrospective matching of names and addresses was used to identify the longitudinal cases embedded in the surveys, where we had interviewed the same householder in the same dwelling on more than one occasion.

We matched survey data to GHA’s records of all works to properties since 2003, along with the dates of completion. The database covers predominantly GHA social rented housing, but also includes owner occupied dwellings within GHA buildings (15.3 % at T1). Through this process, we derived a matched, longitudinal sample of 1933 cases, comprising 9.5 % of all GHA households in our study areas. Figure 1 shows the embedded longitudinal cohort and demonstrates how we constructed the sub-samples for analysis in this paper.

### Health outcomes

We examine differences in self reported health conditions among those who did and did not have housing improvements of each type between T1 and T2. The following long term health conditions, lasting 12 months or more, are reported by respondents at each survey wave:

- Respiratory Health (Breathing problems, asthma, bronchitis)
- Circulatory Health (Heart, high blood pressure, blood circulation problems)
- Digestive Health (Stomach, liver, kidney, digestive problems)
- Long Term Migraine/Headache Condition (Migraine or frequent headaches)
- Long term skin conditions/allergies (Skin conditions/allergies)
- Mental Health (Stress, anxiety or depression)

### Housing improvement data

GHA record housing improvements in the following categories: Central Heating, Front Doors, Windows, Environmental, Fabric Works, Internal Common Works, Lift Replacement and Kitchen, Bathroom & Rewiring. Four of these had a high enough rate of provision among our survey respondents to warrant further analyses: kitchen, bathroom and rewiring; central heating; front doors; and fabric works. Brief synopses of these works are given in Table 2. Kitchen, bathroom and rewiring consists of fully refitted bathrooms and kitchens, including equipment, cupboards, tiling and flooring, plus renewal of all wiring in properties. These can be seen as quality, aesthetic and safety improvements to the home. The occupants have choice about finishings on floors, wooden surrounds, cupboards and tiling. Central heating involves installing or upgrading existing central heating systems, as well as boiler and hot water tank replacement. The type of heating system varied between property types. All properties had existing full or partial heating systems so the results in this paper relate to improvement of systems rather than provision of heating where it did not previously exist. New front doors were to police-approved ‘secured by design’ standards and are viewed primarily as a security improvement, although may also be a warmth improvement if installed as a package of works. Fabric works include a range of external improvements including insulation, cladding, roof renewal and balcony repairs. Fabric works are therefore both aesthetic and warmth improvements.

**Table 2:** Number (%) of respondents experiencing housing improvements

Housing improvement	Description	Number (%) receiving improvement
New kitchen, bathroom & rewiring	New kitchen and bathroom equipment. Tiling. Cupboards. Linoleum flooring.	706 (35.7 %)
	Customer choice of finishings.	
Central Heating	Boiler replacement. New central heating system. New water tank.	374 (18.9 %)
Doors	New ‘secured by design’ front doors.	483 (24.4 %)
Fabric Works	New roof covering. Over-cladding (high-rise). Wall and roof insulation. Rendering or repointing to walls. New gutters and downpipes.	575 (29.1 %)

For each respondent-occupied dwelling, we attached to our survey data information on the type of housing improvement(s) received and date of completion of the works. Table 2 shows the proportion of matched longitudinal respondents who had each type of housing improvement between the two interviews. Different types of works to the same property may be done at different times, not as part of a single improvement package of works.

### Analyses

For the purpose of this analysis we combine three waves of survey data into T1 (before) and T2 (after) as shown in Fig. 1. We have three ‘wave pairings; Wave 1- Wave 2, Wave 2 – Wave 3, and Wave 1 – Wave 3, which are re-coded into T1 and T2.

We undertake analysis in two stages. Firstly we focus on those *with* each of the pre-existing health conditions at T1. To examine the curative or ameliorative effect of housing improvements, we use binary logistic regression to calculate the odds ratio of respondents having the same health condition at T2, dependent on whether or not they experienced each type of housing improvement associated with the health condition (see Table 1) between interviews. For this analysis, we do not have sufficient cases of respondents with allergies or skin conditions at T1 to investigate intervention effects. Nor are there enough cases of respondents with headaches or mental health problems at T1 who subsequently received central heating prior to T2. Thus, for the analysis of improving health, we investigate 12 of the 17 housing improvement pathways identified in Table 1.

Secondly we focus on those *without* each of the health conditions at T1. To examine the scope for a preventative effect of housing improvements we use binary logistic regression to calculate the odds ratios of respondents reporting the condition at T2, dependent on whether or not they experienced each type of housing improvements associated with the health condition in the intervening period (see Table 1). For this analysis of worsening health, we are able to investigate all 17 housing improvement pathways identified in Table 1.

We control for whether or not a respondent lives in a regeneration area due to the potentially different nature of housing improvements in these areas, where large scale demolition is taking place. We also control for gender, age, change in working status or working status at T1 (depending on sample size, in some cases models would not estimate using change in working status so we revert to working status at T1), educational qualifications and citizenship. given that health is strongly influenced by smoking [40] we control for  change in smoking status or smoking status at T1 (depending on sample size, in some cases models would not estimate using change in smoking status so we revert to smoking status at T1). Given that the sample is constructed from three waves of survey data we also control for the wave pairing, due to both differing time intervals and potential contextual effects. However, for mental health we only include Wave 2 – Wave 3 cases as the survey question changed after Wave 1, so we do not include this control in the mental health models. In the preventative analyses we control for the number of long term health conditions reported at T1, so as to adjust for cases of co-morbidity.

We checked for multicollinearity among the explanatory variables firstly using Spearman correlation and then using OLS regression and checking the VIF and tolerance levels. There were no causes for concern. Analyses were undertaken using IBM SPSSv22.

### Ethical approval

The NHS Scotland multicentre research ethics committee approved the study in January 2006.

## Results

Table 3 shows the number of respondents reporting each health condition and mean number of health conditions at T1 and T2 as well as the numbers who recover from or develop conditions over time, based on reporting of conditions at each time point.

**Table 3:** Number (%) of respondents reporting health conditions and mean number of conditions

	T1	T2	Recovered from condition (as % of those with condition at T1)	Developed condition (as % of those without condition at T1)
Respiratory	256 (13.%)	371 (19 %)	126 (49 %)	242 (12 %)
Circulatory	323 (16 %)	443 (23 %)	170 (53 %)	291 (15 %)
Digestive	85 (4 %)	160 (8.%)	65 (77 %)	140 (7 %)
Migraine/headache	122 (6 %)	189 (9 %)	96 (79 %)	160 (8 %)
Skin/allergies	30 (2 %)	135 (6 %)	20 (66 %)	125 (6 %)
Mental health	93 (14 %)	137 (21 %)	42 (45 %)	86 (15 %)
Number of LT health conditions (mean)	0.42 (0.75)	0.67 (0.97)	281 (44.7 %)^a^	564 (28.6 %)^b^

^a^Decreased number as a proportion of those who had one or more conditions at T1

^b^Increased number as a proportion of those who had less than 6 conditions at T1

For all health conditions, a greater proportion of all respondents report problems at T2 than at T1. More people report developing a condition rather than recovering from a health condition over time. The rate of recovery is greater than the rate of developing a condition, due to the different denominator population in each case. The mean number of health conditions reported across the sample has also increased, from 0.42 conditions per respondent at T1 to 0.67 at T2, with more people reporting an increased number of health conditions than a decrease. We have previously reported the growing problem of co-morbidity across our study groups [41].

### Recovery from health conditions

Table 3 shows that between T1 and T2 between 45 % and 79 % of those who reported a long term health condition at T1 did not report it at T2. We tested the effect of specific housing improvements on reporting of health conditions for those who had reported long term health conditions at T1, based on the hypothesised relationships outlined in Table 1.

We test twelve combinations and find two significant relationships between housing improvements and improved health conditions (Tables 4 and 5). Those who had central heating are more likely (OR = 2.63) to have recovered from circulatory conditions between interviews than those who did not have central heating improvements. This is expected based on our conceptual framework as central heating is a warmth improvement and warmth is a key risk factor associated with circulatory health conditions. Although not reaching statistical significance, the effect of fabric works on circulatory health conditions operated in a similar curative direction (OR = 1.60).

**Table 4:** Curative effects: odds ratios (95 % CI) of not reporting health condition at T2, when it was reported at T1 (recovering from condition)

	Respiratory			Circulatory		Digestive
	Fabric works	Central heating	Kitchen, bathroom & rewiring	Fabric works	Central heating	Kitchen, bathroom & rewiring
Intervention	0.95 (0.46,1.95)	0.81 (0.35,1.9)	0.69 (0.34,1.39)	1.60 (0.85,2.98)	2.63 (1.17,5.92)*	0.33 (0.07,1.48)
*Change in smoking behaviour (ref: non-smoker)*						
smoker	0.99 (0.49,2.02)	0.86 (0.38,1.95)	0.75 (0.39,1.42)	1.21 (0.63,2.32)	0.84 (0.38,1.86)	3.3 (0.77,14.17)
stopped smoking	1.15 (0.32,4.11)	1.63 (0.39,6.7)	1.09 (0.36,3.34)	0.86 (0.28,2.67)	2 (0.6,6.72)	3.46 (0.45,26.49)
started smoking	1.18 (0.27,5.16)	0.54 (0.09,3.43)	1.12 (0.3,4.16)	3.01 (0.52,17.58)	1.39 (0.29,6.75)	1.35 (0.07,24.62)
Demolition Area	1.21 (0.51,2.86)	0.87 (0.31,2.44)	1.01 (0.43,2.34)	1.8 (0.88,3.71)	1.44 (0.55,3.74)	1.08 (0.17,6.62)
Female (ref: male)	0.85 (0.44,1.64)	0.75 (0.35,1.61)	0.67 (0.37,1.2)	0.88 (0.5,1.56)	0.65 (0.34,1.27)	2.16 (0.56,8.33)
Over 65 (T1)	2.5 (1.21,5.18)*	1.49 (0.65,3.41)	1.46 (0.75,2.84)	2.15 (1.16,4)*	1.61 (0.76,3.42)	2.98 (0.6,14.67)
Not working (T1)	0.25 (0.05,1.39)	0.23 (0.04,1.35)	0.45 (0.12,1.71)	0.32 (0.09,1.12)	0.39 (0.09,1.68)	1.13 (0.15,8.71)
Educational quals (ref:none) (T1)	0.92 (0.38,2.25)	0.63 (0.2,2.02)	0.84 (0.36,1.99)	0.84 (0.37,1.92)	1.54 (0.58,4.13)	0.88 (0.13,6.18)
British (ref:not british) (T1)	0.75 (0.11,5.28)	2 (0.14,27.66)	1.02 (0.23,4.63)	5.23 (1,27.23)*	2.12 (0.46,9.77)	0.21 (0.02,2.8)
*Wave pairing (ref: Wave 1- Wave 2)*						
Wave 1 - Wave 3	1.14 (0.45,2.88)	0.82 (0.31,2.17)	1.18 (0.53,2.62)	0.42 (0.19,0.89)*	0.46 (0.2,1.05)	0.35 (0.06,2.17)
Wave 2 -Wave 3	0.76 (0.33,1.77)	0.48 (0.16,1.42)	0.7 (0.31,1.57)	0.55 (0.25,1.2)	0.42 (0.15,1.12)	1.24 (0.18,8.7)
Constant	2.52	2.35	1.71	5.94	3.98	2.22
Nagelkerke R2	0.087	0.083	0.07	0.145	0.191	0.231
n	173	132	208	232	173	72

*p<0.05; **P<0.01

**Table 5:** Curative effects: odds ratios (95 % CI) of not reporting health condition at T2, when it was reported at T1 (recovering from condition)

	Headache			Mental health		
	Fabric works	Doors	Kitchen, bathroom & rewiring	Fabric works	Doors	Kitchen, bathroom & rewiring
Intervention	1.47 (0.36,5.97)	0.37 (0.04,3.98)	0.19 (0.04,1.03)	3.55 (1.03,12.23)*	2.35 (0.77,7.15)	1.88 (0.97,3.62)
*Change in smoking behaviour (ref: non-smoker)*				0.38 (0.11,1.36)	2.52 (0.78,8.14)	1.41 (0.67,2.95)
smoker	0.23 (0.06,0.84)*	0.07 (0.01,0.57)*	0.28 (0.08,0.96)*			
stopped smoking	0.83 (0.03,23.7)	0.1 (0,10.34)	0.36 (0.01,12.07)			
started smoking	0.61 (0.03,13.87)	0.04 (0,1.9)	0.49 (0.03,8.58)			
Demolition Area	0.47 (0.11,1.97)	0.61 (0.08,4.55)	0.15 (0.02,0.96)*	1.76 (0.46,6.75)	0.32 (0.1,1.01)	0.17 (0.06,0.46)**
Female (ref: male)	0.91 (0.25,3.39)	2.22 (0.37,13.12)	0.65 (0.2,2.12)	1.42 (0.41,4.92)	0.98 (0.34,2.84)	0 (0,0)*
Over 65 (T1)	2.15 (0.46,10.09)	4.62 (0.44,49.07)	1.92 (0.42,8.73)	10.27 (0.83,126.58)	2.2 (0.47,10.28)	0.32 (0.12,0.82)*
Not working (T1)	3.4 (0.42,27.8)	10.3 (0.6,177.65)	7.01 (0.83,58.96)	0.21 (0.01,3.12)	5.11 (0.42,62.65)	0.26 (0.06,1.17)
Educational quals (ref:none) (T1)	0.87 (0.18,4.17)	4.33 (0.25,74.53)	1.64 (0.34,7.99)	1.16 (0.33,4.04)	0.78 (0.18,3.44)	0.45 (0.14,1.44)
British (ref:not british) (T1)	2.54 (0.23,28.09)	1.74 (0.07,44.92)	4.33 (0.37,50.3)	1.01 (0.1,9.75)	2.42 (0.11,52.82)	1.28 (0.64,2.53)
*Wave pairing (ref: Wave 1- Wave 2)*						
Wave 1 - Wave 3	0.16 (0.02,1.24)	0.52 (0.06,4.2)	0.37 (0.08,1.77)			
Wave 2 -Wave 3	0.23 (0.03,1.61)	0.52 (0.04,7.07)	0.35 (0.07,1.8)			
Constant	4.94	2.58	3.06	11.87	0.44	0.72
Nagelkerke R2	0.228	0.388	0.285	0.254	0.227	0.282
n	80	53	98	64	19	65

*p<0.05; **P<0.01

We also found that those who had fabric works were more likely,to not report mental health problems at T2 which they had previously reported (OR = 3.55), than those who did not have fabric works. This is concurrent with our hypothesised relationship in Table 1 whereby we expect a positive relationship between fabric works and mental health due to improved external appearance of the home, which may affect how residents perceive their home and themselves in status terms. Apart from the housing improvements, the only other factor found to be associated with improved health over time, was being aged over 65 at T1, which increased the odds ratio of recovering from three of the health conditions over time: respiratory health; circulatory conditions; and mental health.

### Prevention of the onset of health conditions

We test 17 combinations and do not find any significant relationships between housing improvements and the onset of health conditions between interviews (Tables 6 and 7). The main factors affecting onset of health conditions in our models are employment and pre-existing health conditions. In the case of four of the health conditions – respiratory health, circulatory conditions, allergies and mental health - those remaining in employment are less likely to develop health conditions (typically OR = 0.16–0.35).. In relation to respiratory health, circulatory health and headaches/migraine, the number of pre existing health conditions is positively associated with the likelihood of developing conditions over time in most of the models (OR = 1.47–2.05) In the respiratory health models, continuing smokers are more likely to develop respiratory health conditions over time (OR = 1.59–1.72). Again, we found that being aged over 65 at T1 was associated with a lower odds ratio of developing three of the health conditions over time: allergies or skin conditions; headaches/migraines; and mental health problems. Being older was however, associated with a higher likelihood of developing a circulatory condition over time.

**Table 6:** Preventative effects: odds ratios (95 % CI) of reporting health condition at T2, when it was not reported at T1 (developing condition)

	Respiratory			Circulatory		Digestive
	Fabric works	Central heating	Kitchen, bathroom & rewiring	Fabric works	Central heating	Kitchen, bathroom & rewiring
Intervention	1.2 (0.84,1.72)	1.25 (0.82,1.91)	1 (0.69,1.44)	1.19 (0.85,1.68)	1.02 (0.69,1.53)	1.05 (0.65,1.69)
*Change in smoking behaviour (ref: non-smoker)*						
smoker	1.72 (1.17,2.53)**	1.31 (0.85,2)	1.59 (1.11,2.26)*	0.62 (0.42,0.91)*	0.7 (0.47,1.04)	1.5 (0.95,2.39)
stopped smoking	1.8 (0.96,3.37)	1.43 (0.72,2.84)	1.37 (0.74,2.54)	1.25 (0.7,2.22)	1.07 (0.56,2.06)	1.56 (0.72,3.38)
started smoking	1.33 (0.61,2.92)	1.22 (0.53,2.83)	1.01 (0.47,2.16)	0.67 (0.32,1.41)	0.72 (0.31,1.66)	1.27 (0.51,3.18)
Demolition Area	1.37 (0.93,2.03)	1.07 (0.63,1.8)	1.27 (0.84,1.92)	0.68 (0.45,1.02)	0.98 (0.6,1.6)	1.33 (0.77,2.28)
Female (ref: male)	0.95 (0.67,1.34)	0.85 (0.59,1.24)	0.78 (0.57,1.06)	0.6 (0.44,0.84)**	0.52 (0.36,0.74)**	0.88 (0.58,1.32)
Over 65 (T1)	0.97 (0.65,1.45)	0.88 (0.57,1.37)	0.95 (0.65,1.39)	1.32 (0.92,1.91)	1.12 (0.75,1.67)	0.67 (0.4,1.1)
*Change in employment status (ref: remain in unemployment)/not working (T1)*						2.22 (1.13,4.38)*
remain in employment	0.25 (0.11,0.56)**	0.35 (0.16,0.76)**	0.31 (0.16,0.62)**	0.19 (0.08,0.42)**	0.16 (0.06,0.37)**	
move out of employment	0.85 (0.41,1.76)	0.65 (0.28,1.53)	0.78 (0.4,1.51)	0.57 (0.27,1.2)	0.93 (0.46,1.91)	
gain employment	0.61 (0.25,1.47)	0.69 (0.3,1.62)	0.57 (0.25,1.3)	0.22 (0.07,0.72)*	0.21 (0.06,0.7)*	
Educational quals (ref:none) (T1)	0.54 (0.32,0.92)*	0.58 (0.33,1.05)	0.66 (0.41,1.06)	0.72 (0.45,1.13)	0.58 (0.34,0.99)*	0.92 (0.53,1.61)
British (ref:not british) (T1)	0.53 (0.28,0.99)*	0.54 (0.25,1.14)	0.45 (0.24,0.83)*	0.62 (0.34,1.15)	0.2 (0.08,0.5)**	0.42 (0.18,0.98)*
*Wave pairing (ref: Wave 1- Wave 2)*						
Wave 1 - Wave 3	2.58 (1.62,4.11)**	2.79 (1.72,4.52)**	2.7 (1.78,4.08)**	1.7 (1.11,2.61)*	2.39 (1.53,3.74)**	2.78 (1.56,4.97)**
Wave 2 -Wave 3	1.49 (0.91,2.45)	1.83 (1.03,3.25)*	1.5 (0.95,2.38)	1.15 (0.74,1.79)	1.71 (1.02,2.87)*	2.24 (1.22,4.12)**
Number of long term health problems (T1)	1.57 (1.18,2.08)**	2.05 (1.48,2.83)**	1.65 (1.27,2.14)**	1.51 (1.12,2.03)**	1.07 (0.77,1.5)	1.3 (0.99,1.7)
Constant	0.07	0.07	0.07	0.07	0.05	0.03
Nagelkerke R2	0.123	0.12	0.12	0.146	0.18	0.067
n	1276	969	1400	1186	928	1517

*p<0.05; **P<0.01

**Table 7:** Preventative effects: odds ratios (95 % CI) of reporting health condition at T2, when it was not reported at T1 (developing condition)

	Headache				Allergies			Mental health			
	Fabric works	Central heating	Doors	Kitchen, bathroom & rewiring	Fabric works	Central heating	Kitchen, bathroom & rewiring	Fabric works	Central heating	Doors	Kitchen, bathroom & rewiring
Intervention	0.99 (0.64,1.53)	0.89 (0.51,1.55)	0.87 (0.48,1.57)	0.98 (0.63,1.54)	0.85 (0.52,1.38)	0.84 (0.48,1.48)	1.22 (0.75,1.98)	0.64 (0.34,1.21)	1.01 (0.26,3.97)	0.6 (0.12,3.11)	0.76 (0.4,1.44)
*Change in smoking behaviour (ref: non-smoker)*											
smoker	0.72 (0.46,1.15)	0.69 (0.4,1.2)	0.75 (0.43,1.32)	0.7 (0.45,1.1)	1.15 (0.7,1.89)	0.9 (0.51,1.57)	0.95 (0.6,1.51)	1.39 (0.7,2.77)	1.1 (0.44,2.76)	1.88 (0.71,4.99)	1.26 (0.64,2.48)
stopped smoking	0.59 (0.25,1.39)	0.91 (0.39,2.1)	0.79 (0.31,2.03)	0.67 (0.31,1.44)	0.72 (0.28,1.89)	0.46 (0.13,1.56)	0.57 (0.22,1.47)	1.01 (0.3,3.36)	0.43 (0.05,3.76)		1.02 (0.3,3.43)
started smoking	0.73 (0.29,1.84)	0.96 (0.37,2.47)	0.75 (0.26,2.14)	0.81 (0.36,1.83)	1.34 (0.54,3.35)	1.76 (0.71,4.33)	1.36 (0.61,3.03)	1.21 (0.24,6.19)	1.9 (0.33,10.91)		1.46 (0.37,5.73)
Demolition Area	1.81 (1.15,2.84)*	1.53 (0.78,3.02)	2.62 (1.38,5)**	1.64 (1,2.67)*	1.62 (1,2.63)	1.51 (0.79,2.89)	1.48 (0.88,2.49)	1.41 (0.67,2.95)	2.22 (0.43,11.58)	0.56 (0.06,5.35)	1.11 (0.52,2.37)
Female (ref: male)	1.77 (1.14,2.75)*	1.52 (0.92,2.5)	1.76 (1.03,2.99)*	1.64 (1.09,2.47)*	1.06 (0.68,1.65)	0.77 (0.47,1.26)	0.93 (0.62,1.39)	1.88 (0.97,3.62)	1.65 (0.65,4.25)	1.95 (0.69,5.53)	2.35 (1.21,4.53)*
Over 65 (T1)	0.16 (0.08,0.32)**	0.19 (0.09,0.4)**	0.15 (0.07,0.34)**	0.17 (0.09,0.32)**	0.56 (0.31,1.01)	0.49 (0.27,0.91)*	0.49 (0.29,0.85)*	0.17 (0.06,0.46)**	0.21 (0.07,0.63)**	0.25 (0.06,0.98)*	0.2 (0.08,0.5)**
*Change in employment status (ref: remain in unemployment)/not working (T1)*			1.48 (0.77,2.84)								
remain in employment	0.69 (0.37,1.29)	0.56 (0.26,1.22)		0.68 (0.37,1.25)	0.94 (0.48,1.84)	0.32 (0.12,0.86)*	0.6 (0.29,1.21)	0.32 (0.12,0.82)*	0.08 (0.01,0.61)*	0.17 (0.03,0.83)*	0.3 (0.12,0.78)*
move out of employment	0.21 (0.05,0.87)*	0.62 (0.21,1.82)		0.35 (0.12,1)	1.02 (0.42,2.49)	0.32 (0.08,1.38)	0.78 (0.32,1.87)	0.26 (0.06,1.17)	0.24 (0.03,2.05)	0.17 (0.02,1.45)	0.2 (0.04,0.91)*
gain employment	0.28 (0.09,0.93)*	0.36 (0.11,1.2)		0.24 (0.07,0.78)*	0.64 (0.22,1.86)	0.16 (0.02,1.18)	0.5 (0.17,1.41)	0.45 (0.14,1.44)	0.48 (0.09,2.56)	0.31 (0.03,2.82)	0.38 (0.12,1.22)
Educational quals (ref:none) (T1)	0.87 (0.52,1.46)	0.79 (0.41,1.51)	0.82 (0.43,1.58)	0.77 (0.46,1.29)	1.22 (0.71,2.09)	1.08 (0.56,2.1)	0.95 (0.56,1.61)	1.28 (0.64,2.53)	0.97 (0.28,3.39)	1.57 (0.55,4.5)	1.22 (0.62,2.39)
British (ref:not british) (T1)	0.58 (0.31,1.1)	0.52 (0.22,1.24)	0.38 (0.15,0.98)*	0.58 (0.31,1.08)	0.93 (0.48,1.8)	0.26 (0.08,0.81)*	0.66 (0.34,1.29)	0.72 (0.3,1.75)	0.11 (0.01,1.2)		0.66 (0.27,1.58)
*Wave pairing (ref: Wave 1- Wave 2)*				0 (0,0)**							
Wave 1 - Wave 3	1.9 (1.08,3.33)*	2.88 (1.5,5.5)**	2.49 (1.36,4.56)**	2.36 (1.4,3.96)**	1.25 (0.7,2.23)	1.42 (0.78,2.6)	1.3 (0.77,2.19)				
Wave 2 -Wave 3	1.79 (1.03,3.1)*	2.54 (1.23,5.23)*	2 (0.9,4.45)	2.02 (1.18,3.46)*	0.97 (0.54,1.73)	1.1 (0.53,2.28)	1.21 (0.71,2.08)				
Number of long term health problems (T1)	1.6 (1.21,2.13)**	1.35 (0.95,1.91)	1.44 (1,2.08)*	1.55 (1.2,2.02)**	1.14 (0.84,1.54)	1.01 (0.72,1.41)	1.03 (0.78,1.36)				
Constant	0.02	0.02	0.03	0.02	0.05	0.02	0.04	0.05	0.02	0.02	0.05
Nagelkerke R2	0.122	0.106	0.123	0.116	0.026	0.066	0.027	0.143	0.202	0.18	0.141
n	1340	1041	949	1510	1398	1087	1581	381	234	174	398

*p<0.05; **P<0.01

The effect of survey wave is significant in several of the onset of health conditions models. This could be because where there was a longer time between interviews, as is the case for Wave 1 to Wave 3 (5 years) and Wave 2 to Wave 3 (3 years) relative to Wave 1 to Wave 2 (2 years) individuals are more likely to develop conditions than over a shorter time period. Alternatively it might reflect a general worsening of the health of the population over time so that in 2011 (Wave 2–3 and Wave 1–3) respondents are more likely to report health conditions that those reported in 2008 (Wave 1–2). This would concur with our general observation of worsening morbidity over time in our study group.

## Discussion

We have examined self-reported health conditions before and after four types of housing improvements. One of the main contributions of our work is to examine these conditions alongside disaggregating the package of housing works into specific types of improvement; many previous studies consider all housing improvement works together, either as a ‘retrofit’ or as a warmth intervention Given long established links between poor housing quality and health problems it can be expected that improvements to housing may lead to improvements in health. However, the evidence directly linking housing improvements to health improvement is limited and somewhat contradictory, in part due to the difficulties in undertaking longitudinal studies both before and after housing improvements, and with a sufficient follow-up interval. If poor housing is a causal factor for health conditions, then it can be expected that as well as potentially ameliorating existing health conditions (consistent with previous research that has found that housing improvements have a more noticeable effect when targeted at those in ill-health) improvements to housing conditions may also mitigate against developing poor health conditions.

We therefore examined health conditions before and after housing improvements both for those with pre-existing health conditions and those without pre-existing health conditions, compared to those with the same health status but who did not experience housing improvements in the same time period. This seems worthwhile given that housing improvements might work in one of two ways, as curative or preventive, and appropriate to our study population which has high and rising rates of morbidity [9]. It is also to our knowledge the first time a housing intervention study has examined the treatment group in this way.

In general, we find more indications of the curative effects of housing improvements than of their preventative effects. We hypothesised 17 pathways between the four housing improvements and six health conditions based on existing knowledge of housing-related risk factors for the conditions in question (see Table 1). In our first set of analyses, we found evidence in support of three of these pathways: two warmth interventions – fabric works and central heating – were associated with a higher likelihood of recovery from circulatory conditions; fabric works were also associated with a higher likelihood of recovery from a mental health condition (anxiety, stress or depression). The first of these findings is in accord with previous studies and adds to a sparse evidence base. The Cochrane Review found only two studies that reported the effects of warmth interventions on circulatory conditions, both reporting beneficial effects: one of these reported a beneficial, curative effect on blood pressure [28]; the other reported beneficial preventative effects on heart disease and hypertension [19] – we did not find evidence for the preventative effect, but for a curative effect. The second finding on the curative effect of fabric works on mental health conditions adds to a conflicting evidence base but supports our earlier findings relating to the effect of fabric works on the SF-12 measure of mental health [10]. Of those studies that used mental health scales as the outcome measure, more found a positive impact of warmth interventions [17, 38, 42] than not [16, 19]. Where self-reported mental illness was used as the outcome, a beneficial effect of housing warmth interventions was reported, as in our study [11].

### Limitations

The poor model fit in our models suggests that we do not adequately include all factors affecting health conditions and that the overall contribution of housing improvements is limited. Below we discuss some of the limitations of the study.

There are several key elements which are not measured in our study. We have no measure of the quality of homes prior to the intervention, so the degree of housing quality gain is not measured. This may explain why in some cases we do not find relationships as expected. For example, in other contexts studies have focused on the heating of homes which had not had a heating system before; in our case, the intervention may be the replacement of an unsatisfactory but nonetheless operational system, so controlling for the existing quality may have demonstrated an effect only where there was substantial quality improvement. We also have no measure of the severity of the health conditions, which means that we might not have picked up on cases where symptoms may have been alleviated, but not entirely cured. Alternatively those who did not have conditions may have developed health conditions in both groups, but they may be less severe among the intervention group and we would not have detected this. Although we did ask respondents to self report whether their conditions had improved, the response rates were too low for analysis. The time elapsed since interventions and the length of time living in the house prior to interventions are also potentially crucial elements which we have not considered here. Whilst we have controlled for socio-demographic characteristics, deprived populations experience many other disruptive events in their lives over short time periods, which can affect their health and which we have not been able to take into account here.

There are other ways in which the analysis for a study such as ours could be set up. Although the improvements are not prioritised by individual need, it may be that those properties eligible or programmed for each type of improvements are those in the worst condition. This may mean that the occupants are more susceptible to developing health conditions or less able to recover so that, despite housing improvements, health is no different among those who have received them due to living in poorer conditions for a length of time. In order to consider this, we would need both better measures of housing conditions for properties receiving and not receiving housing improvements, as well as better measures of the severity and duration of the health conditions of dwelling occupants.

The small sample sizes in some cases mean that the power of our models to find significant effects is restricted. This increases our chances of a type II error, or false negative results. In this study this applies mainly to the small sample sizes in Tables 4 and 5, modelling the curative effects of housing improvements. There are effect sizes which may be considered substantive, but which are not statistically significant and this highlights a need for larger studies of these particular health conditions and housing improvements. Those which might warrant further study include: the effect of fabric works on circulatory health and headaches; kitchen, bathroom and rewiring on digestive conditions; and the effects of doors and new kitchens, bathrooms and rewiring on mental health. When looking at the preventative effects in Tables 6 and 7, the sample sizes are larger so we would expect significant effects to be detected. Furthermore the effect sizes are generally small so it seems less likely that we are not finding substantive effects due to limited statistical power. Nevertheless, some of the relationships may warrant further investigation including: fabric works on respiratory and circulatory conditions; central heating on respiratory conditions and kitchen, bathroom and rewiring on allergies. Given that we provide a conceptual basis for each pathway examined, we have not corrected for multiple comparisons to avoid further risk of type II errors, although this does increase the chance of a type I error.

It may be that different population sub-groups respond differently to housing interventions and also that there are both positive and negative effects of some housing interventions on health. For example, older people may suffer negative effects of disruption from the improvement works and women are more likely to suffer depression in damp homes [13, 43] so may also benefit more from reduced dampness. There is also evidence to suggest that insulation can reduce ventilation leading to adverse health consequences [44]. So both individual differences and the counteracting effects of housing improvements may be masked by our current approach to analysis and more detailed analysis of subgroups in future research may highlight different relationships.

Our study is a form of natural experiment with a complex intervention [45] , where we are studying interventions without any control over the pattern of provision. Whilst it is advantageous to use natural experiments to look at public health interventions [46] there are also weaknesses. In this case, the combination of interventions (housing improvement works) is non-random in ways we do not fully understand; historic practices of housing allocations and property maintenance may mean that certain types of people receive certain combinations of works at this time, while others do not, thus compromising our ability to test their potential effects on the general population. On the other hand, the fact that we can know more about the distinct nature and combinations of housing improvement works is an advantage over many other studies, where housing improvements are largely unknown in character or crudely lumped together as ‘warmth’ or ‘retrofitting’ works [4].

It could be argued that we cannot expect to see changes in an individual’s health, attributed to housing improvements, over relatively short timescales, given they may have been living in poor housing with poor health for a considerable amount of time, despite our period of study being longer than many. Evidence suggests that childhood housing conditions can affect health in adults, regardless of the current housing situation [3]. On this basis, we could call into question the assumption that we might be able to attribute changes in an individual’s health to housing improvements over short time periods, and instead argue that the purpose of housing improvements is to reduce the prevalence of health conditions at a population level in the long-term. Therefore, although controlled experimental design is usually heralded as being more robust for evaluating the effects of interventions on health outcomes, this may not be true when the expected benefits are at the population level rather than being an immediate cure for an individual health problem.

## Conclusion

In many other studies, it is not always clear whether the effect of the housing intervention is curative or preventive, as all that is usually reported are the comparative health statuses post-intervention, unless a study is focused only on those with a health particular condition, which is sometimes the case. The fact that we found evidence for some curative effects of housing improvements, but no evidence for their preventative effects is therefore important as it indicates which of these types of impact is the more likely, at least for a deprived and relatively unhealthy population group. It also provides support for two of the main conclusions of the recent Cochrane review of housing improvements and health, namely that health gain is most likely where housing improvements are targeted at those in most need, and that short term health impacts from improvement programmes delivered across an entire area (as is the case in Glasgow) are less clear [4].

## References

[1] Walsh D, McCartney G, McCullough S, van der Pol M, Buchanan D, Jones R (2013). Exploring potential reasons for Glasgow’s ‘excess’ mortality: results of a three city survey of Glasgow, Liverpool and Manchester. A report by the Glasgow Centre for Population Health, NHS Health Scotland and the University of Aberdeen.

[2] Wilkinson D. Poor housing and ill health: A summary of research evidence. The Scottish Office Central Research unit: Housing Research Branch. Crown Copyright. 1999; 2009.

[3] British Medical Association (2003). Housing and health: building for the future.

[4] Thomson H, Thomas S, Sellstrom E, Petticrew M (2013). Housing improvements for health and associated socio-economic outcomes.

[5] Howden-Chapman P, Matheson A, Crane J, Vigger H, Cunningham M, Blakely T (2007). Effect of insulating existing houses on health inequality: cluster randomised study in the community. BMJ.

[6] Hanlon P, Walsh D, Whyte B. Let Glasgow Flourish. Glasgow Centre for Population Health/. 2006; (http://www.understandingglasgow.com/assets/0000/4811/LetGlasgowFlourish_full.pdf) Accessed: 4/12/14.

[7] Leyland AH, Dundas R, McLoone P, Boddy FA (2007). Inequalities in Mortality in Scotland, 1981–2001. MRC Social and Public Health Sciences Unit Occasional Paper no. 16.

[8] McCarron P, Davey Smith G, Womersley J. Deprivation and mortality in Glasgow: changes from 1980 to 1992. BMJ. 1994; 309. doi: http://dx.doi.org/10.1136/bmj.309.69671: 481. 10.1136/bmj.309.6967.1481 PMC2541610 7804053

[9] Egan M, Tannahill C, Bond L, Kearns A, Mason P. Health outcomes over time Glasgow: GoWell/GCPH.

[10] Curl A, Kearns A, Mason P, Egan M, Tannahil C, Ellaway A. Physical and mental health outcomes following housing improvements: evidence from the GoWell Study. J Epidemiol Community Health. 2014; ISSN 0143-005X (doi:10.1136/jech-2014-204064) (Early Online Publication). 10.1136/jech-2014-204064 25205160

[11] Shortt N, Rugkåsa J (2007). “The walls were so damp and cold” fuel poverty and ill health in Northern Ireland: Results from a housing intervention. Health Place.

[12] Platt S, Martin C, Hunt S. Damp housing, mould growth and symptomatic health state. BMJ. 1989;298. 10.1136/bmj.298.6689.1673 PMC1836778 2503174

[13] Hyndman SJ (1990). Housing dampness and health amongst British Bengalis in East London. Soc Sci Med.

[14] Garrett M, Rayment P, Hooper MA, Abramson MJ, Hooper BM. Indoor airborne fungal spores, house dampness and associations with environmental factors and respiratory health in children. Clin Exp Allergy. 1998;28:459–67. 10.1046/j.1365-2222.1998.00255.x 9641573

[15] Peat J, Dickerson J, Li J (1998). Effects of damp and mould in the home on respiratory health: a review of the literature. Allergy.

[16] Barton A, Basham M, Foy C, Buckingham K, Somerville M, on behalf of the Torbay Healthy Housing Group (2007). The Watcombe Housing Study: the short term effect of improving housing conditions on the health of residents. J Epidemiol Community Health.

[17] Howden-Chapman P, Pierse N, Nicholls S, Gillepsie-Bennet J, Vigger H, Cunningham M (2008). Effects of home heating on asthma in community dwelling children: randomised control trial. BMJ.

[18] Osman LM, Ayres JG, Garden C, Reglisz K, Lyo J, Douglas JG (2010). A randomised trial of home energy efficiency improvement in the homes of elderly COPD patients. Eur Respir J.

[19] Platt S, Mitchell R, Walker J, Hopton J, Petticrew M, Corbett J (2007). The Scottish Executive Central Heating Programme: assessing impacts on health. Research Findings 239.

[20] Woodfine L, Neal RD, Bruce N, Edwards RT, Linck P, Mullock L (2011). Enhancing ventilation in homes of children with asthma: Pragmatic randomised control trial. Br J Gen Pract.

[21] Braubach M, Heinen D, Dame J (2008). Preliminary results of the WHO Frankfurt housing intervention project.

[22] Hopton J, Hunt S (1996). The health effects of improvements to housing: a longitudinal study. Hous Stud.

[23] Somerville M, Mackenzie I, Owen P, Miles D (2000). Housing and health: does installing heating in their homes improve the health of children with asthma?. Public Health.

[24] Iverson M, Bach E, Lundqvist GR (1986). Health and comfort changes among tenants after retrofitting of their housing. Environ Int.

[25] Kearns A, Petticrew M, Mason P, Whitley E (2006). The effects of social housing on health and wellbeing: initial findings from the SHARP study. Research from Communities Scotland. Report No 75.

[26] Ambrose P. A drop in the ocean: the health gain from the Central Stepney SRB in the context of national health inequalities. University of Brighton: Brighton: The Health and Social Policy Research Centre; 2000. http://eprints.brighton.ac.uk/10751/. Accessed 4/12/14.

[27] Blackman T, Harvey J, Lawrence M, Simon A (2001). Neighbourhood renewal and health: evidence from a local case study. Health Place.

[28] Lloyd EL, McCormack C, McKever M, Syme MT (2008). The effect of improving the thermal quality of cold housing on blod pressure and general health: a research note. J Epidemiol Community Health.

[29] Walker J, Mitchell R, Petticrew M, Platt S (2008). The effects on health of a publicly funded domestic heating programme: a prospective controlled study. J Epidemiol Community Health.

[30] Molnar A, Adany R, Adam B, Gulis G, Kosa K (2010). Helath impact assessment and evaluation of a Roma housing project in Hungary. Health Place.

[31] Fuller-Thompson E, Hulchanski JD, Hwang A (2000). The housing/health relationship: what do we know?. Rev Environ Health.

[32] Gilbertson JM, Grimsley M, Green G (2012). Psychosocial routes from housing investment to health gain. Evidence from England’s home energy efficiency scheme. For the Warm Front Study Group. Energy Policy.

[33] Liddell C (2013). Tackling fuel poverty: mental health impacts and why these exist. Presentation at the IEA-EEA Rountable on the Health and Well-being Impacts of energy.

[34] International Energy Agency (2014). Capturing the multiple benefits of energy efficiency.

[35] Thomson H, Morrison D, Petticrew M (2007). The health impacts of housing-led regeneration: a prospective controlled study. J Epidemiol Community Health.

[36] Critchley R, Gilberston J, Green G, Grimsley M (2004). Housing investment and health in Liverpool.

[37] Wells N (2000). Housing quality and women’s mental health: a 3 wave longitudinal study (conference proceeding). European Network for Housing Research Conference. Housing in the 21st Century: Fragmentation and Reorientation.

[38] Allen T (2005). Evaluation of the housing for healthier hearts project April 2003-March 2005.

[39] Thomas R, Evans S, Huxley P, Gately C, Rogers A (2005). Housing improvements and self-reported mental distress among council estate residents. Soc Sci Med.

[40] U.S. Department of Health and Human Services (2014). The health consequences of smoking: 50 years of progress.

[41] GoWell Progress for People and Places: Monitoring Change in Glasgow’s Communities Glasgow: GoWell/GCPH.

[42] Allen T (2005). Private sector housing improvement in the UK and the chronically ill: implications for collaborative working. Hous Stud.

[43] Brown GW, Harris T (1978). Social origins of depression: a study of psychiatric disorder in women.

[44] Gens A, Hurley JF, Tuomisto JT, Friedrich R (2014). Health impacts due to personal exposure to fine particles caused by insulation of residential buildings in Europe. Atmos Environ.

[45] Petticrew M, Cummins S, Ferrell C, Findlay A, Higgins C, Hoy C (2005). Natural experiments: an underused tool for public health?. Public Health.

[46] Craig P, Cooper C, Gunnell D, Haw S, Lawson K, Macintyre S, et al. Using natural experiments to evaluate population health interventions: new Medical Research Council guidance. J Epidemiol Community Health. 2012;66(12):1182–6. 10.1136/jech-2011-200375 PMC3796763 22577181

